# Supplementary material for: Comparison of perceived masticatory ability in completely edentulous patients treated with thermoplastic complete denture versus single implant-retained mandibular overdenture: a single-center prospective observational study
Source: PeerJ. 2024 Jul 5;12:e17670. doi: 10.7717/peerj.17670 (PMC11229681; doi:10.7717/peerj.17670)
Supplement: Supplemental Information 2 [file peerj-12-17670-s002.docx]

STROBE Statement—checklist of items that should be included in reports of observational studies

|  | Item No. | Recommendation | Page  No. | Relevant text from manuscript |
| --- | --- | --- | --- | --- |
| **Title and abstract** | 1 | (*a*) Indicate the study’s design with a commonly used term in the title or the abstract | 2 | Perceived masticatory ability evaluation in completely edentulous patients with thermoplastic complete denture versus single implant-retained mandibular overdenture |
|  |  | (*b*) Provide in the abstract an informative and balanced summary of what was done and what was found | 2 | Methods: This study was conducted in the outpatient Prosthodontic Clinic, Faculty of Dental Medicine, Al-Azhar University.Cairo,Egypt. The perceived masticatory ability (PrMA) was evaluated in 45 completely edentulous patients. Each patient received a thermoplastic PMMA complete denture (Polyan IC TM Bredent GmbH & Co.KG, Germany). The PrMA was evaluated at one month and after six months of denture use. For each patient an immediate loading single implant was placed in the mid-symphyseal area and the denture was modified, then, the PrMA was evaluated again after one month and after six months. Data were collected and statistically analyzed with SPSS@ V25 to assess the changes in perceived masticatory ability.  Results: The PrMA improved after six months of thermoplastic conventional denture use but was not statistically significant (p= 0.405). PrMA increased significantly after a single implant placement after one and six months (p<0.001) of the overdenture use compared to the conventional denture. The PrMA insignificantly improved (p= 0.397) after six months of the single implant retained overdenture use. |
| Introduction | | | |  |
| Background/rationale | 2 | Explain the scientific background and rationale for the investigation being reported | 3 | One of the main objectives of prosthodontic rehabilitation is to restore and maintain oral function especially the effective masticatory function |
| Objectives | 3 | State specific objectives, including any prespecified hypotheses | 5 | Up to our knowledge , there was no study evaluating the perceived masticatory ability (assessed subjectively) among completely edentulous patients rehabilitated with a thermoplastic acrylic denture before and after the single implant placement for each patient to assess the changes in the perceived masticatory ability with single implant placement. Thus, the null hypothesis was that placing a single implant to retain a complete mandibular thermoplastic denture would not affect the perceived masticatory ability. |
| Methods | | | |  |
| Study design | 4 | Present key elements of study design early in the paper | 6 | A total of 50 patients who had lost all of their teeth were chosen randomly from those who sought treatment at the outpatient clinic of the Department of Prosthodontics. Five patients withdrew from the study , so only 45 patients were evaluated. The group consisted of 21 males and 24 females, with an average age range of 44-59 years (mean age 50.4 ± 4.77 years) |
| Setting | 5 | Describe the setting, locations, and relevant dates, including periods of recruitment, exposure, follow-up, and data collection | 6 | This study was conducted at the Faculty of Dental Medicine, Al-Azhar University, Egypt, using a prospective study design. The study was conducted for a duration of 17 months, spanning from April 2022 to August 2023. |
| Participants | 6 | (*a*) *Cohort study*—Give the eligibility criteria, and the sources and methods of selection of participants. Describe methods of follow-up  *Case-control study*—Give the eligibility criteria, and the sources and methods of case ascertainment and control selection. Give the rationale for the choice of cases and controls  *Cross-sectional study*—Give the eligibility criteria, and the sources and methods of selection of participants | 6 | All patients included in the study were free of any psychiatric problems or movement disorders. Patients who have previously had temporomandibular problems, including Myofacial Pain Dysfunction Syndrome (MPDS), trismus, trauma, TMJ dislocation, and ankylosis, were not included in the study. Furthermore, those with compromised oral diseases , local lesions, xerostomia and resorbed or flabby ridges were not included. |
|  |  | (*b*) *Cohort study*—For matched studies, give matching criteria and number of exposed and unexposed  *Case-control study*—For matched studies, give matching criteria and the number of controls per case |  |  |
| Variables | 7 | Clearly define all outcomes, exposures, predictors, potential confounders, and effect modifiers. Give diagnostic criteria, if applicable | 6 | All patients received a new thermoplastic PMMA conventional complete denture (Polyan IC TM bredent GmbH & Co.KG, Germany), with even occlusion, and free from discomfort.  The new complete dentures were delivered, and the perceived masticatory ability (PrMA) was evaluated after one month of denture placement without pain or discomfort |
| Data sources/ measurement | 8* | For each variable of interest, give sources of data and details of methods of assessment (measurement). Describe comparability of assessment methods if there is more than one group | *7* | *The subjective approach of evaluating masticatory ability was assessing the PrMA. The measurement was conducted using a perceived difficulty of chewing (PDC) index score devised by Khalifa et al,(Khalifa et al. 2013) in which respondents were queried about the level of difficulty they had while chewing fifteen commonly consumed hard and soft foods. The scoring of this index is based on (PDC) scale, with a range of scores from 0 (indicating very easy chewing) to 5 (indicating very difficult chewing that is actively avoided). A total number of zero signifies a very easy chewing and satisfactory conditions, whereas a total score of 75 signifies adverse conditions and the most difficult chewing.* |
| Bias | 9 | Describe any efforts to address potential sources of bias | 7 | The second measurement was conducted six months following the conventional thermplastic denture placement, as recommended by Goiato (Goiato et al. 2010; Goiato et al. 2008) It was proposed that a minimum of five months was required to adequately assess patient adaptability and functional capacity with new complete dentures. |
| Study size | 10 | Explain how the study size was arrived at | 6 | Previous studies have determined that a sample size of 40 cases is sufficient to conduct the research with a statistical power of 0.80, a confidence interval of 0.95, and an alpha level. 0.05.(Albert et al. 2003; Goiato et al. 2010; Mohamed 2008; Tatematsu et al. 2004) A higher sample size calculation was considered (n=50). To allow for the possibility of edentulous participants dropping out due to illness, death, or difficulty with the research protocol. |

Continued on next page

| Quantitative variables | 11 | Explain how quantitative variables were handled in the analyses. If applicable, describe which groupings were chosen and why | 8 | For each patient, The PrMA was measured after after one month of single implat-retained mandibular overdenture placement. The final measurment was conducted after six months of single implat-retained mandibular overdenture placement. |
| --- | --- | --- | --- | --- |
| Statistical methods | 12 | (*a*) Describe all statistical methods, including those used to control for confounding | 8 | The data were collected, and the statistical analysis was conducted using IBM SPSS Statistics V25 software (Armonk, NY: IBM Corp) with a significant level of 0.05 for all tests. For continuous data, they were tested for normality by the Shapiro-Wilk test. Quantitative data were expressed as range (minimum and maximum), mean, standard deviation and median. Descriptive statistics of mean and standard deviation were reported. Mann Whitney test was used to compare two groups for not normally distributed quantitative variables while Kruskal Wallis test was used to compare different groups for not normally distributed quantitative variables. while Friedman test For abnormally distributed quantitative variables, to compare between more than two periods or stages. The post-hoc paired comparison was done using the Wilcoxon signed rank test. Significance of the obtained results was judged at the 5% level.  The null hypothesis is rejected and there is strong evidence that there is a difference between the groups. When the null hypothesis has been rejected, it is possible to test which groups are different. The est (pairwise comparison) is output for this purpose. |
|  |  | (*b*) Describe any methods used to examine subgroups and interactions |  |  |
|  |  | (*c*) Explain how missing data were addressed |  |  |
|  |  | (*d*) *Cohort study*—If applicable, explain how loss to follow-up was addressed  *Case-control study*—If applicable, explain how matching of cases and controls was addressed  *Cross-sectional study*—If applicable, describe analytical methods taking account of sampling strategy |  |  |
|  |  | (*e*) Describe any sensitivity analyses |  |  |
| Results | | | | |
| Participants | 13* | (a) Report numbers of individuals at each stage of study—eg numbers potentially eligible, examined for eligibility, confirmed eligible, included in the study, completing follow-up, and analysed | 9 | The sample included 50 completely edentulous patients who were randomly selected, only 45 patients were evaluated ( one patient was released for health reasons, four patients declined to continue with the research). There were 21 male and 24 female patients Table (1). The mean age of the selected patients was 50.46 years ranging from 44 years to 59 years. |
|  |  | (b) Give reasons for non-participation at each stage | 9 | only 45 patients were evaluated ( one patient was released for health reasons, four patients declined to continue with the research) |
|  |  | (c) Consider use of a flow diagram |  |  |
| Descriptive data | 14* | (a) Give characteristics of study participants (eg demographic, clinical, social) and information on exposures and potential confounders | 9 | There were 21 male and 24 female patients Table (1). The mean age of the selected patients was 50.46 years ranging from 44 years to 59 years. |
|  |  | (b) Indicate number of participants with missing data for each variable of interest |  |  |
|  |  | (c) *Cohort study*—Summarise follow-up time (eg, average and total amount) | 9 | The mean and standard deviation of the perceived masticatory ability measurements at different intervals were shown in Table (2) |
| Outcome data | 15* | *Cohort study*—Report numbers of outcome events or summary measures over time | *9* | *The perceived masticatory ability for each participant was obtained by collecting the perceived masticatory ability score (from 0 to 5) of each food. The mean value for perceived masticatory ability was 37.8 ±10.5 at the time of one month after the new denture placement. After six months of denture placement, the mean value of perceived masticatory ability was 36.3 ± 10.3. The mean value for perceived masticatory ability was 28.6 ±8.4 at the time of one month after the single implant placement. After six months of single implant placement, the mean value of perceived masticatory ability was 26.9 ± 8.5. (Table 2) .* |
|  |  | *Case-control study—*Report numbers in each exposure category, or summary measures of exposure |  |  |
|  |  | *Cross-sectional study—*Report numbers of outcome events or summary measures |  |  |
| Main results | 16 | (*a*) Give unadjusted estimates and, if applicable, confounder-adjusted estimates and their precision (eg, 95% confidence interval). Make clear which confounders were adjusted for and why they were included | 9 | The multiple comparison between different mean measurements of PrMA at different intervals (Table 5) showed that there was no statistical difference in perceived masticatory ability recorded after one month of denture insertion and perceived masticatory ability recorded after six months of denture insertion. [ P > 0.05]. The perceived masticatory ability increased considerably with mid-symphyseal single Implant placement and there was a highly statistically significant difference between the perceived masticatory ability recorded before and after single implant placement.[ P < 0.05].  Regarding the age, the study sample was further allocated based on age range into three subgroups: (1) less than 47 (n=13), (2) from 47 - 52 (n=14), and (3) more than 52 (n=18). |
|  |  | (*b*) Report category boundaries when continuous variables were categorized |  |  |
|  |  | (*c*) If relevant, consider translating estimates of relative risk into absolute risk for a meaningful time period |  |  |
| Other analyses | 17 | Report other analyses done—eg analyses of subgroups and interactions, and sensitivity analyses | 10 | Regarding the age, the study sample was further allocated based on age range into three subgroups: (1) less than 47 (n=13), (2) from 47 - 52 (n=14), and (3) more than 52 (n=18).  The Mann Whitney test ( Table 6) was used to test the effect of gender on the perceived masticatory ability at different intervals. The results showed no statistically significant effect of gender on the PrMA at different intervals. The Kruskal Wallis test ( Table 7) was used to test the effect of different age groups on the perceived masticatory ability at different intervals. The results showed no statistically significant effect of gender on the PrMA at different intervals. |

Continued on next page

| Discussion | | | | |
| --- | --- | --- | --- | --- |
| Key results | 18 | Summarise key results with reference to study objectives | 12 | Regarding the age, the study sample was further allocated based on age range into three subgroups: (1) less than 47 (n=13), (2) from 47 - 52 (n=14), and (3) more than 52 (n=18).  The Mann Whitney test ( Table 6) was used to test the effect of gender on the perceived masticatory ability at different intervals. The results showed no statistically significant effect of gender on the PrMA at different intervals. The Kruskal Wallis test ( Table 7) was used to test the effect of different age groups on the perceived masticatory ability at different intervals. The results showed no statistically significant effect of gender on the PrMA at different intervals. |
| Limitations | 19 | Discuss limitations of the study, taking into account sources of potential bias or imprecision. Discuss both direction and magnitude of any potential bias | 13 | The limitation of this study includes that the alveolar ridge height and denture retention, which could influence the results, were not investigated. Also, the association between self-assessed masticatory ability (SAMA) and psychological status should be considered as the results of Roohafza et al (Roohafza et al. 2016) study provide evidence that participants with a higher score of depression, anxiety, and stress suffer lower masticatory ability.  It is also important to emphasize the diagnostic factor and previous planning before denture construction. The human factors in planning and technical performance are decisive for the success of rehabilitation. |
| Interpretation | 20 | Give a cautious overall interpretation of results considering objectives, limitations, multiplicity of analyses, results from similar studies, and other relevant evidence | 12 | Even when the comparison is made between perceived masticatory ability evaluated after six months of conventional denture use with that assessed one month after a single implant retained mandibular over denture, there was a highly significant difference. (Table 3) This finding demonstrates the considerable enhancement following the insertion of a single implant. Also the masticatory function significantly improves after mandibular implant overdenture treatment. Most studies on implant treatment and oral function showed a significant improvement of the objective masticatory performance in the mandibular overdenture. (Fontijn-Tekamp et al. 2004)  The study of Rocha et al. (Rocha et al. 2023), evaluated the masticatory function objectively also confirmed the importance of using a single implant to improve the masticatory function for a completely edentulous patient. |
| Generalisability | 21 | Discuss the generalisability (external validity) of the study results | 13 | Within the limitations of the present study, a significant improvement in perceived masticatory ability was observed in completely edentulous patients rehabilitated with single implant-retained mandibular overdentures. |
| Other information | |  | | |
| Funding | 22 | Give the source of funding and the role of the funders for the present study and, if applicable, for the original study on which the present article is based | 14 | The authors declare there was no funding for this work. |

*Give information separately for cases and controls in case-control studies and, if applicable, for exposed and unexposed groups in cohort and cross-sectional studies.

**Note:** An Explanation and Elaboration article discusses each checklist item and gives methodological background and published examples of transparent reporting. The STROBE checklist is best used in conjunction with this article (freely available on the Web sites of PLoS Medicine at http://www.plosmedicine.org/, Annals of Internal Medicine at http://www.annals.org/, and Epidemiology at http://www.epidem.com/). Information on the STROBE Initiative is available at www.strobe-statement.org.
